# Patient-Specific iPSC-Based Models of Huntington’s Disease as a Tool to Study Store-Operated Calcium Entry Drug Targeting

**DOI:** 10.3389/fphar.2018.00696

**Published:** 2018-06-29

**Authors:** Vladimir Vigont, Evgeny Nekrasov, Alexey Shalygin, Konstantin Gusev, Sergey Klushnikov, Sergey Illarioshkin, Maria Lagarkova, Sergey L. Kiselev, Elena Kaznacheyeva

**Affiliations:** ^1^Institute of Cytology, Russian Academy of Sciences, Saint Petersburg, Russia; ^2^Vavilov Institute of General Genetics, Russian Academy of Sciences, Moscow, Russia; ^3^Scientific Center of Neurology, Russian Academy of Medical Sciences, Moscow, Russia; ^4^Federal Research and Clinical Center of Physical-Chemical Medicine, Moscow, Russia

**Keywords:** store-operated calcium channels, SOC, Huntington’s disease, neurodegeneration, EVP4593, iPS cells, CRAC channels

## Abstract

Neurodegenerative pathologies are among the most serious and socially significant problems of modern medicine, along with cardiovascular and oncological diseases. Several attempts have been made to prevent neuronal death using novel drugs targeted to the cell calcium signaling machinery, but the lack of adequate models for screening markedly impairs the development of relevant drugs. A potential breakthrough in this field is offered by the models of hereditary neurodegenerative pathologies based on endogenous expression of mutant proteins in neurons differentiated from patient-specific induced pluripotent stem cells (iPSCs). Here, we study specific features of store-operated calcium entry (SOCE) using an iPSCs-based model of Huntington’s disease (HD) and analyze the pharmacological effects of a specific drug targeted to the calcium channels. We show that SOCE in gamma aminobutyric acid-ergic striatal medium spiny neurons (GABA MSNs) was mediated by currents through at least two different channel groups, *I*_CRAC_ and *I*_SOC_. Both of these groups were upregulated in HD neurons compared with the wild-type neurons. Thapsigargin-induced intracellular calcium store depletion in GABA MSNs resulted in predominant activation of either *I*_CRAC_ or *I*_SOC_. The potential anti-HD drug EVP4593, which was previously shown to have neuroprotective activity in different HD models, affected both *I*_CRAC_ and *I*_SOC_.

## Introduction

Neurodegenerative disorders represent one of the most difficult challenges for modern medicine and healthcare: because their etiology and pathogenesis remain largely obscure. It has been shown, however, that these disorders are associated with significant dysregulation of calcium homeostasis (Wojda et al., 2008; Bezprozvanny, 2009; Berridge, 2010; Riazantseva et al., 2012; Imamura et al., 2016; Alzheimer’s Association Calcium Hypothesis Workgroup, 2017; Surmeier et al., 2017), and the same is also true of cardiac pathologies (Røe et al., 2015; Wagner et al., 2015) and allergies (Yang et al., 2013).

Calcium as a second messenger controls a number of intracellular processes such as gene expression, cell differentiation, proliferation, and apoptosis (Clapham, 2007). One of the most common types of calcium influx in cells is SOC entry (SOCE), which plays an important role in calcium homeostasis in both non-excitable (Poggioli and Putney, 1982) and neuronal cells (Bouron et al., 2005). Activation of SOC channels results from intracellular calcium store depletion triggered by an increased level of inositol 1,4,5-trisphosphate (IP_3_) in the cytosol and subsequent activation of the IP_3_ receptor (Kaznacheyeva et al., 2001). Impotently, alterations in SOCE are intimately linked with Alzheimer’s disease (Ryazantseva et al., 2013, 2017; Tong et al., 2016) and HD (Wu et al., 2011; Czeredys et al., 2017; Kolobkova et al., 2017).

Huntington’s disease is caused by an autosomal dominant mutation in the gene encoding a protein called huntingtin. HD manifests in neural loss predominantly of GABA MSNs in striatum (Vonsattel and DiFiglia, 1998; Kolobkova et al., 2017). In HD, the mutated huntingtin is characterized by polyglutamine expansion within N-terminus region of this protein. Normally the length of polyglutamine tract does not exceed 35 glutamine residues, whereas longer tracts are associated with HD pathology.

Our studies on dysregulation of calcium homeostasis in different HD cell models such as human neuroblastoma SK-N-SH (Wu et al., 2011; Vigont et al., 2014), mouse neuroblastoma Neuro-2a (Vigont et al., 2015), and primary culture of mouse striatal neurons (Vigont et al., 2015) have shown that neuronal SOC channels play a crucial role in HD pathogenesis and can be considered as a potential target for medical treatment. This approach requires the development of a “personalized” platform for the screening of new-generation neuroprotective drugs.

The recently developed technology for deriving patient-specific iPSCs, which offer an effective model for drug screening, has become a real breakthrough in modern pharmacology (Ishida et al., 2016; Mungenast et al., 2016). We have previously designed a new iPSCs-based model of HD, and experiments with it have shown that SOCE in HD-specific GABA MSNs (HD GABA MSNs) is pathologically enhanced, compared to wild-type GABA MSNs (WT GABA MSNs), and that this enhancement is closely correlated with the expression of mutant huntingtin in the cells (Nekrasov et al., 2016).

It is known that SOCE depends on two different channel groups, *I*_CRAC_ and *I*_SOC_ (Bugaj et al., 2005; Parekh and Putney, 2005; Ambudkar et al., 2007; Vig and Kinet, 2007; Skopin et al., 2013; Shalygin et al., 2015), with the set of SOCE channels varying between different cell types. Both *I*_CRAC_ and *I*_SOC_ could serve as a specific target for medical treatment of HD. In this study, we used the above iPSC-based model of HD in an attempt to find out which SOC channel types are upregulated in HD-specific human neurons. We also addressed the question of possible discrimination of these channels types by using a potential anti-HD drug and SOCE inhibitor EVP4593 (Wu et al., 2011).

## Materials and Methods

### Cells

Experiments were performed with patient-specific striatal GABA MSNs, which were generated using the protocol described in details previously (Nekrasov et al., 2016). Briefly, cultures of primary dermal fibroblasts were established from skin biopsies of three female HD patients and two healthy donors. Then, fibroblasts were transduced with lentiviral vectors LeGO-hOCT4, LeGO-hSOX2, LeGO-hc-Myc, and LeGO-hKLF4 and cultured to form iPSCs. To validate the pluripotency potential of iPSCs, we confirmed the expression of pluripotency markers (Oct4, SSEA-4) as well as ability of iPSCs to form teratomas iPSCs and their differentiation capacity into cells of all three germ layers. The iPSCs derived from HD patients and healthy donors and human embryonic stem cell line were effectively differentiated into GABA MSNs which were attested by the expression of specific neuronal marker TUBB3 and specific GABA MSN marker DARPP-32, as well as by the ability of these neurons to form spines and synapses and their voltage-sensitivity. As a result, we obtained three HD-specific neuronal lines – iPSHD11 (Q40), iPSHD22 (Q47), and iPSHD34 (Q42) – and three wild-type (control) neuronal lines: iPSRG2L, endo-iPS12, and hESM01. All these six GABA MSN lines were used for electrophysiological studies.

### Electrophysiological Studies

Ion currents were recorded using the whole-cell patch-clamp technique (Hamill and Sakmann, 1981). The measurements were made with an Axopatch 200B amplifier (Axon Instruments, United States). The microelectrode resistance was 5–10 MΩ; the series resistance was not compensated but continuously monitored throughout the experiment, with its values being in the range of 10–25 MΩ. The signal was enhanced and filtered with an internal 2-pole Bessel filter (section frequency 5000 Hz) and digitized at 5000 Hz using an AD convertor plate (L-Card, Russia). During the recording of integral currents, the membrane potential initially held at −40 mV was periodically (every 5 s) decreased to −100 mV for 30 ms, then gradually raised to 100 mV at a rate of 1 mV/ms, and then returned to −40 mV. Measurements were made at 0.5-mV intervals. The recorded currents were normalized relative to cell capacitance (6–20 pF). The traces recorded prior to current activation were used as templates for leak subtraction. The pipette solution contained (in mM) 125 CsCl, 10 EGTA-Cs, 30 HEPES-Cs, 4.5 CaCl_2_, 1.5 MgCl_2_, 4 Mg-ATP, and 0.4 Na-GTP pH 7.3 adjusted with CsOH. The extracellular solution contained (in mM) 140 NMDG-Asp, 10 BaCl_2_, 30 HEPES-Cs, 0.01 nifedipine, and pH 7.3 adjusted with CsOH. Currents were evoked by application of 1 μM thapsigargin to the external solution. To suppress SOC currents, 100 nM EVP4593 was used. All chemicals were from Sigma-Aldrich (United States). Statistical comparisons were made using one-way ANOVA with Bonferroni correction (normality and equal variances were also checked by the Shapiro–Wilk and Levene tests, respectively). The results were considered statistically significant at *p* < 0.05.

## Results

### SOCE in GABA MSNs Is Mediated by at Least Two Different Types of Channels

Two main protein families contributing to SOCE are Orai (Hoth and Penner, 1992; Parekh and Putney, 2005; Vig and Kinet, 2007) and TRPC (Ambudkar et al., 2007; Salido et al., 2009; Smyth et al., 2010). The Orai1 protein forms highly selective calcium-permeable CRAC channels with a strong inward rectification of current–voltage relationship (*I*/*V*) curve. Data on the role of TRPC proteins in SOCE are somewhat controversial and these data concern mainly TRPC1 (Ambudkar et al., 2007; Salido et al., 2009; Skopin et al., 2013) and TRPC3 (Kaznacheyeva et al., 2007; Alkhani et al., 2014). We have demonstrated this role for the TRPC1 protein in our previous experiments on different cell models of HD (Wu et al., 2011; Vigont et al., 2015). The corresponding SOCE channels are characterized by a nearly linear *I*/*V* curve and relatively low selectivity for calcium. It has been shown that drug targeting to calcium channels results in significant alterations of electrophysiological signals (Vegara-Meseguer et al., 2015; Ryazantseva et al., 2017). To gain a deeper insight into the mechanism of drug targeting, it is important to discriminate between currents maintained by CRAC and TRPC channels.

**FIGURE 1:**
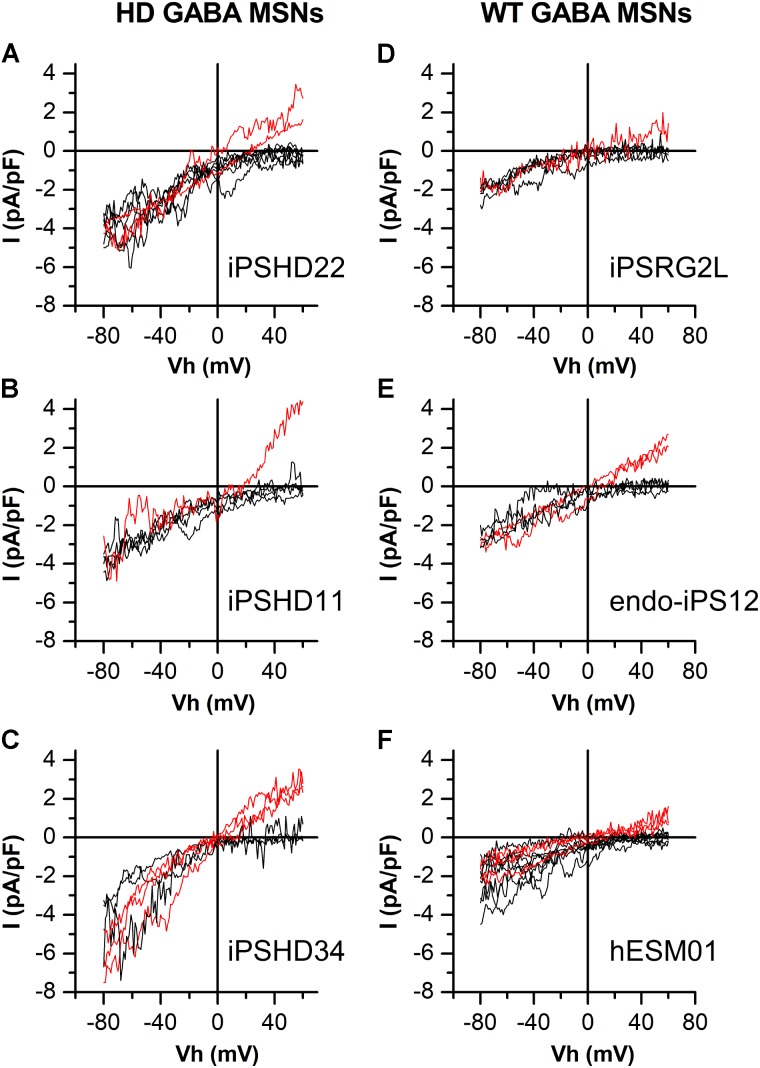
Different types of store-operated calcium currents in patient-specific GABA MSNs. **(A–F)** Current/voltage relationships (*I*/*V* curves) for currents evoked by passive calcium store depletion with 1-μM thapsigargin in HD GABA MSNs: **(A)** iPSHD22, **(B)** iPSHD11, and **(C)** iPSHD34 and in WT GABA MSNs: **(D)** iPSRG2L, **(E)** endo-iPS12, and **(F)** hESM01. Measurements were made when the currents reached a maximum. Each curve represents the result of an individual experiment. Black curves refer to currents with strong inward rectification; red curves refer to currents with a relatively linear *I*/*V* relationship and low reversal potential.

In this study, electrophysiological experiments were performed to investigate calcium entry through SOC channels in both HD GABA MSNs (**Figures 1A–C**) and WT GABA MSNs (**Figures 1D–F**). SOCE was evoked by application of 1-μM thapsigargin which blocked the SERCA pump in the endoplasmic reticulum membrane and thereby caused intracellular calcium store depletion, with consequent activation of SOC channels in the plasma membrane. We have previously succeeded in attributing specific currents to certain channel types by knocking down individual channel subunits (Vigont et al., 2015). As follows from **Figures 1A–C**, whole-cell recordings from our new iPSC-based model of HD allowed spontaneous discrimination between different types of SOC currents. We observed currents with strong inward rectification which is a characteristic of CRAC channels (*I*_CRAC_, black curves). In some experiments, we detected currents with an obvious outward component and a nearly linear *I*/*V* curve which is typical for low selective TRPC channels (*I*_SOC_, red curves). The same was true of neurons generated from iPSCs established from healthy individuals and normal ES cell line (WT GABA MSNs) (**Figures 1D–F**). The observed effect was reproducible in different cell lines generated from individual HD patients and healthy individuals. The frequency of recording *I*_SOC_ was about 33% for WT GABA MSNs (**Figures 1D–F**) and about 28% for HD GABA MSNs (**Figures 1A–C**), whereas *I*_CRAC_ was recorded approximately twice as frequently (**Table 1**).

**Table 1:** Frequency of *I*_CRAC_ and *I*_SOC_ appearance in human GABA MSNs.

Cell line	*I*_SOC_, %	*I*_CRAC_, %
iPSHD22 (HD)	25	75
iPSHD11 (HD)	20	80
iPSHD34 (HD)	50	50
iPSRG2L (WT)	20	80
endo-iPS 12 (WT)	40	60
hESM01 (WT)	27	73

These results indicate that calcium store depletion in GABA MSNs usually results in strong predominant activation of one of the two SOC channel types in these cells (i.e., either *I*_CRAC_ or *I*_SOC_). The *I*/*V* curves of *I*_CRAC_ and *I*_SOC_ at negative potentials were similar within each cell line but their behaviors at positive potentials were significantly different.

By using transcriptome analysis, it has been previously shown that the expression of Orai genes, members of the TRPC family, and regulatory STIM genes did not vary significantly between cell lines (Nekrasov et al., 2016).

### Both *I*_CRAC_ and *I*_SOC_ Are Pathologically Enhanced in HD GABA MSNs

We have previously shown that SOCE is pathologically increased in HD cell models (Wu et al., 2011; Vigont et al., 2014, 2015). Namely, it was twice as large in HD GABA MSNs than that in WT GABA MSNs (Nekrasov et al., 2016). Here, a detailed electrophysiological analysis of spontaneously differentiated *I*_CRAC_ and *I*_SOC_ showed that both components of SOCE were enhanced in HD GABA MSN. In order to find out whether both SOC channel types are upregulated in HD-specific neurons, we divided all the currents from **Figure 1** into two groups: *I*_CRAC_ and *I*_SOC_.

**FIGURE 2:**
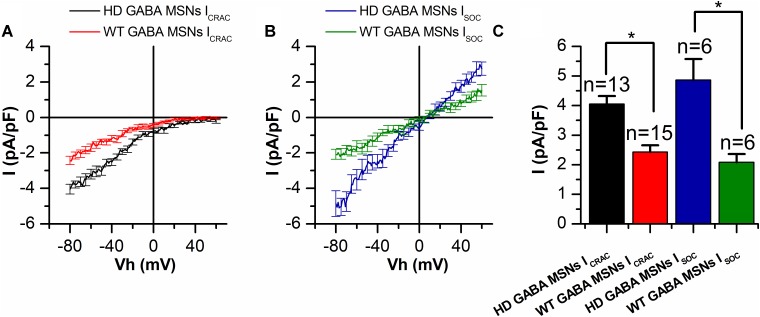
Both *I*_CRAC_ and *I*_SOC_ amplitudes are increased in HD GABA MSNs. **(A,B)** Average current/voltage relationships (*I*/*V* curves) for currents evoked by passive calcium store depletion with 1-μM thapsigargin for **(A)** *I*_CRAC_ in HD GABA MSNs (black curve) and in WT GABA MSNs (red curve) and **(B)** *I*_SOC_ in HD GABA MSNs (blue curve) and in WT GABA MSNs (green curve). Measurements were made when the currents reached a maximum. Each curve represents the average of several experiments from **Figure 1** and plotted as mean ± SEM. The number of experiments is shown in **(C)** above the bars. **(C)** Average amplitudes (normalized to cell capacity) for *I*_CRAC_ in HD GABA MSNs (black bar) and in WT GABA MSNs (red bar) and *I*_SOC_ in HD GABA MSNs (blue bar) and in WT GABA MSNs (green bar). For all groups, current amplitude was determined at a test potential of –80 mV and plotted as mean ± SEM. An asterisk indicates that differences in current amplitudes are statistically significant (*p* < 0.05).

In whole-cell recordings, the amplitude of thapsigargin-induced *I*_CRAC_ inward currents in HD GABA MSNs was found to be significantly enhanced reaching a maximum of −4.05 ± 0.27 pA/pF, compared with −2.32 ± 0.25 pA/pF in control WT GABA MSNs at −80 mV holding potential (**Figures 2A,C**). The same was true of the amplitude of thapsigargin-induced *I*_SOC_ currents: −4.98 ± 0.87 vs. −1.83 ± 0.24 pA/pF, respectively (**Figures 2B,C**).

### Both *I*_CRAC_ and *I*_SOC_ Are Sensitive to the Potential Anti-HD Drug EVP4593

The EVP4593 compound was initially described as an inhibitor of activation of NF-κB signaling pathway (Tobe et al., 2003). Our previous studies have shown that EVP4593 can also attenuate SOCE in different cell models of HD including patient-specific GABA MSNs (Wu et al., 2011; Vigont et al., 2015; Nekrasov et al., 2016).

**FIGURE 3:**
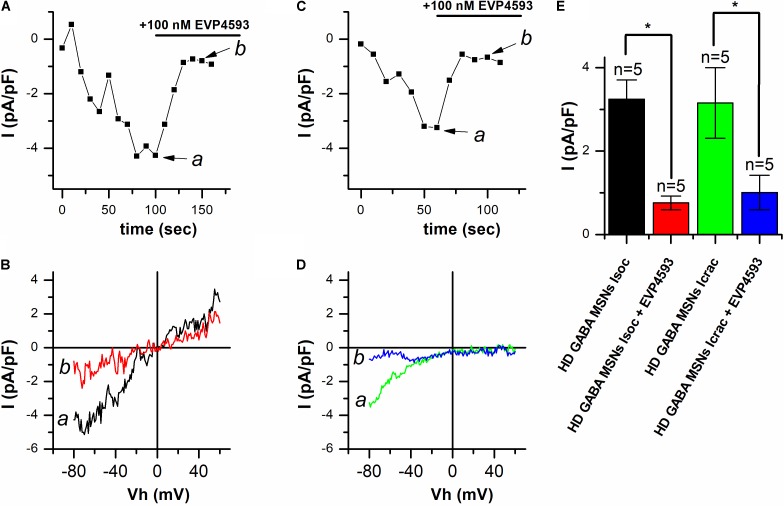
EVP4593 decreases both *I*_CRAC_ and *I*_SOC_ in HD GABA MSNs. **(A,C)** Amplitudes of thapsigargin-induced store-operated calcium currents in HD GABA MSNs at a test potential of –80 mV plotted as a function of time in case of **(A)** *I*_CRAC_ activation and **(C)** *I*_SOC_ activation. The period of 100 nM EVP4593 application is indicated by black line above the graphs. Data from representative experiments are shown. **(B,D)** Current/voltage relationships (*I*/*V* curves) of thapsigargin-induced store-operated calcium currents in HD GABA MSNs in case of **(B)** *I*_CRAC_ activation and **(D)** *I*_SOC_ activation [data from the same experiments as in **(A)** and **(C)**]. Black curves (marked *a*) show the maximum level of current before EVP4593 application; red curves (marked *b*), the steady state level of current after blocking by EVP4593. Ramps corresponding to curves *a* and *b* for *I*_CRAC_ and *I*_SOC_ are indicated by arrows in **(A)** and **(C)**, respectively. **(E)** Average amplitudes (normalized to cell capacity) for *I*_CRAC_ in HD GABA MSNs before (black bar) and after application of 100 nM EVP4593 (red bar) and *I*_SOC_ in HD GABA MSNs before (green bar) and after application of 100 nM EVP4593 (blue bar). For all groups, current amplitude was determined at a test potential of –80 mV and plotted as mean ± SEM. An asterisk indicates that differences in current amplitudes are statistically significant (*p* < 0.05).

In our previous studies in the presence of thapsigargin, usually a superposition of currents through different types of SOC channels (Vigont et al., 2014, 2015) was observed. As a result, we were not able to determine which type of SOC channels is affected by EVP4593. Here, we observed the predominant activation of one type of SOC channels, namely, *I*_CRAC_ or *I*_SOC_. We used the opportunity to discriminate between the currents (see **Figure 1**) in an attempt to find out whether EVP4593 can decrease both *I*_CRAC_ and *I*_SOC_ or only one of them. To this end, thapsigargin-induced responses in HD GABA MSNs were recorded using the whole-cell patch clamp technique, with 100 nM EVP4593 being applied after the currents reached a maximum. The results showed that EVP4593 effectively decreased SOCE in case of both *I*_CRAC_ (**Figure 3A**) and *I*_SOC_ (**Figure 3C**) without having any effect on the shape of *I*/*V* curves being observed after particular current blocking (**Figures 3B,D**). On average, application of 100 nM EVP 4593 to HD GABA MSNs decreased the amplitude of *I*_CRAC_ from −3.24 ± 0.46 to −0.76 ± 0.16 pA/pF (*n* = 5) and the amplitude of *I*_SOC_ from −3.15 ± 0.85 to −1.01 ± 0.41 pA/pF (*n* = 5) (**Figure 3E**).

Thus, we conclude that (i) EVP4593 can significantly affect both *I*_CRAC_ and *I*_SOC_ in HD GABA MSNs and that (ii) the previously observed neuroprotective effect of EVP4593 may be partly explained by its activity toward CRAC and TRPC channels.

## Discussion

It is well known that SOCE in different types of cells can be mediated not only by Orai-based CRAC channels but also by TRPC channels (containing mostly TRPC1 and TRPC3) (Prakriya and Lewis, 2015). Moreover, it has been reported that calcium entry through CRAC channels can regulate TRPC1 membrane trafficking (Cheng et al., 2011). The results of our studies indicate that intracellular calcium store depletion in GABA MSNs not only triggers calcium entry though highly selective CRAC channels but could also lead to an activation of relatively non-selective calcium-permeable channels that might be formed by TRPC subunits. We have previously reported that SOCE in HD cell models such as human neuroblastoma SK-N-SH cells (Vigont et al., 2014) and mouse neuroblastoma Neuro-2A cells (Vigont et al., 2015) is mediated by at least two different types of SOC channels. The *I*/*V* curve of the integral SOC current usually demonstrated a superposition of *I*/*V* curves of individual currents. The predominant activation of one channel type has been observed only in case of suppression of SOCE activator protein STIM1 (Vigont et al., 2014) or knockdown of the main subunit of the other channel (Vigont et al., 2015). Here, we have shown that activation of *I*_CRAC_ and *I*_SOC_ in patient-specific GABA MSNs can occur independently in both HD and WT neurons (**Figure 1**). This can be partly explained by possible heterogeneity of our iPSCs-based model of HD. Significant variations in electrophysiology were recently observed in neurons differentiated from over 100 iPSC lines (Schwartzentruber et al., 2018) Thus, individual neurons may display significant variability in *I*_CRAC_ and *I*_SOC_ activation even in case of absence the differences in the expression of Orai, TRPC, and STIM genes in neuronal populations as we observed. It should be noted, however, that we have previously observed such an effect in human carcinoma A431 cells, where calcium store depletion led to activation of a superposition of highly selective *I*_CRAC_ (with strong inward rectification) and low selective, relatively linear *I*_SOC_ as well as to a separate activation of *I*_CRAC_ or *I*_SOC_ alone (Gusev et al., 2003). Our data indicate that in most cases, calcium store depletion in human GABA MSNs results in strong predominant activation of either *I*_CRAC_ or *I*_SOC_, but it cannot be excluded that some of the SOC currents may represent a superposition of *I*_CRAC_ and *I*_SOC_.

The results presented above show that both types of store-operated calcium channels (*I*_CRAC_ and *I*_SOC_) are equally upregulated in HD GABA MSNs, compared with wild-type neurons (**Figure 2**). It should be noted that in the human neuroblastoma SK-N-SH cell model of HD used in our previous experiments, the increased SOCE was not connected with alterations in the expression of channel-forming proteins. In particular, the level of TRPC1 expression in SK-N-SH cells transfected with mutant huntingtin proved to remain constant (Wu et al., 2011). Subsequent experiments with this model also have not revealed any upregulation in the expression of proteins responsible for SOCE, including Orai1 and STIM1 (unpublished data). This allowed us to suggest that the increase in SOCE is most likely caused by changes in channel activation due to alterations in store depletion machinery. This suggestion is in good agreement with the data that mutant huntingtin could potentiate the receptor for IP_3_ and cause alterations in calcium content in the endoplasmic reticulum (Tang et al., 2003). Since STIM1 and STIM2 proteins regulating SOC channels are sensitive to calcium concentration in the lumen of endoplasmic reticulum (Dziadek and Johnstone, 2007), more complete store depletion can result in stronger activation of these proteins and, therefore, in increasing calcium entry through the SOC channels. It should also be noted that STIM1 and STIM2 have different affinity to calcium ions. Thus, different activation profiles of SOC channels can be explained by differences in the content of calcium in the endoplasmic reticulum, complete or incomplete store depletion, and relative levels of STIM1 and STIM2 expression in the cells (Shalygin et al., 2015).

The dramatic increase in SOCE observed in HD GABA MSNs expressing low-repeat mutant huntingtin suggests that SOCE dysregulation may be one of the key factors of pathology progression and may also be responsible for other calcium alterations, such as mitochondrial calcium overload. The results of previous studies show that the potential anti-HD drug EVP4593 affects channels that contain TRPC1 as a subunit but not channels formed by TRPC1 alone (Wu et al., 2011). TRPC1 participates in important neuronal processes related to synaptic transmission and plasticity (Bröker-Lai et al., 2017). The involvement of TRPC1 in pathological calcium influx in the cells is confirmed by data that TRPC1 knockdown or TRPC channel blocking by 2-aminoethoxydiphenyl borate (2-APB) protects murine hippocampal cell line HT22 against glutamate toxicity (Narayanan et al., 2014). In addition to SOC inhibitory activity, EVP4593 has also been shown to have a neuroprotective effect in fly and mouse HD models (Wu et al., 2011). In addition, it significantly and dose-dependently reduced MG132-induced cell death in the HD GABA MSN model, with the highest efficiency at 100 nM (Nekrasov et al., 2016). Our data suggest that the neuroprotective effect of EVP4593 can be connected with SOCE inhibition, since a similar effect was observed after molecular knockdown of TRPC1 in SK-N-SH cells (Wu et al., 2011).

Trying to discriminate between different SOC channels with respect to their pharmacological potential for HD treatment, we addressed the question as to whether EVP4593 can affect both *I*_CRAC_ and *I*_SOC_ or only one of these channel types in HD GABA MSNs. As mentioned above, in HD models used in previous studies, we usually observed a superposition of different SOC currents (Vigont et al., 2014, 2015). Furthermore, when we suppressed one of the main channel subunits in order to discriminate between the channels, the amplitude of current proved to be too low to obtain conclusive evidence for EVP4593 efficiency. Here, we observed strong predominant activation of a certain SOC channel type, which provided a possibility to test whether EVP4593 could decrease both *I*_CRAC_ and *I*_SOC_ or only one type of these currents. The results showed that EVP4593 in a high nanomolar concentration decreased both *I*_CRAC_ and *I*_SOC_ in HD GABA MSNs (**Figure 3**). The direct molecular target of EVP4593 is still unknown, but our recent data showing that EVP4593 equally affects different SOC channels in the same cell lines suggest that its target is likely to be a shared SOCE regulatory protein (e.g., one of STIM proteins) rather than a channel subunit.

The new patient-specific iPSCs-based HD model is a promising platform for both basic research and drug screening. Further research on this model will lead to the better understanding of the molecular mechanisms of neurodegeneration and help to find novel targets for medical treatment.

## Ethics Statement

The study was approved by the local ethical committee of Scientific Center of Neurology (Moscow, Russia). All patients signed informed consent before skin biopsy procedure.

## Author Contributions

SLK and EK conceived the study. VV, AS, SI, SLK, ML, and EK analyzed the data and wrote the manuscript. VV, AS, and KG performed the calcium currents electrophysiological recordings. EN, SK, and ML performed the generation of cells and expression analysis.

## Conflict of Interest Statement

The authors declare that the research was conducted in the absence of any commercial or financial relationships that could be construed as a potential conflict of interest.

## Abbreviations

CRAC: calcium release-activated calcium (channels)
GABA MSNs: gamma aminobutyric acid-ergic medium spiny neurons
HD: Huntington’s disease
iPSCs: induced pluripotent stem cells
SOC(E): store-operated calcium (entry)
